# Transdiagnostic alterations in neural emotion regulation circuits – neural substrates of cognitive reappraisal in patients with depression and post-traumatic stress disorder

**DOI:** 10.1186/s12888-022-03780-y

**Published:** 2022-03-08

**Authors:** Micha Keller, Raul Mendoza-Quiñones, Amaray Cabrera Muñoz, Jorge Iglesias-Fuster, Anette Valdés Virués, Mikhail Zvyagintsev, J. Christopher Edgar, Jana Zweerings, Klaus Mathiak

**Affiliations:** 1 Department of Psychiatry, Psychotherapy and Psychosomatics, School of Medicine, RWTH Aachen University Hospital, Pauwelsstr. 30, 52074 Aachen, Germany; 2 Department of Cognitive Neuroscience, Cuban Center for Neuroscience, Havana, Cuba; 3 Department of Radiology, Children’s Hospital of Philadelphia, Philadelphia, USA; 4 JARA-Brain, Research Center Jülich, Jülich, Germany

**Keywords:** Depression, Post-traumatic stress disorder, Emotion regulation, Cognitive reappraisal, Lateral PFC

## Abstract

**Background:**

Impaired cognitive reappraisal, associated with the social functioning and well-being of patients affected by mood or anxiety disorders, is characterized by distinct neural activation patterns across clinical populations. To date, studies dedicated to identifying common and distinct neural activation profiles need to be clarified. The aim of the present study was to investigate transdiagnostic differences and commonalities in brain activation patterns during reappraisal-mediated downregulation of emotions.

**Methods:**

Cognitive reappraisal of negative images was contrasted with maintaining emotions during a control viewing condition. Brain activation in 35 patients with major depressive disorder (MDD), 20 patients with post-traumatic stress disorder (PTSD), and 34 healthy controls (HC) during cognitive reappraisal was compared. Moreover, the neural circuitry of emotion regulation in these clinical populations was examined using seed-to-voxel and voxel-to-voxel functional connectivity analyses.

**Results:**

Whole-brain fMRI analyses showed less right-lateralized activation of the inferior, middle, and superior frontal gyrus during cognitive reappraisal compared to viewing of negative images in MDD and PTSD patients compared to HCs. Right IFG activation was negatively correlated with the severity of anxiety and depressive symptomatology. In addition, increased seed-to-voxel connectivity of the right IFG as well as increased voxel-to-voxel connectivity was observed in PTSD patients compared to HCs and MDD patients.

**Conclusions:**

FMRI results therefore suggested a common deficit of depression and anxiety symptomatology reflected by reduced activation in right IFG during cognitive reappraisal as well as diagnosis specific effects in patients with PTSD based on seed-to-voxel and voxel-to-voxel connectivity showing an overactive and hyperconnected salience network. Findings highlight the role of transdiagnostic research to identify disorder specific brain patterns as well as patterns common across disorders.

**Supplementary Information:**

The online version contains supplementary material available at 10.1186/s12888-022-03780-y.

## Background

The identification of neural mechanisms related to key clinical features in psychiatric disorders may help to tailor individualized brain-based training programs to treat cognitive deficits. For instance, the investigation of the neural underpinnings of emotion dysregulation and sustained negative affect as core features of anxiety and mood disorders may help identify neural targets for novel treatments such as real-time fMRI neurofeedback or transcranial direct current stimulation [1–3]. Current studies on emotion regulation in psychiatric disorders such as major depressive disorder (MDD) and post-traumatic stress disorder (PTSD) often investigate the use of cognitive reappraisal, an antecedent focused emotion regulation strategy, that entails the attempt to reinterpret an emotion-eliciting situation in order to change its emotional impact [4–9].

### Neural substrates of cognitive reappraisal

Investigations of the neural bases of emotion regulation are critical to understanding the relation between a reduced capacity to use cognitive reappraisal and the severity of depressive symptoms [10]. Cognitive reappraisal involves the down-regulation of negative emotions and is associated with enhanced activation of medial and lateral prefrontal regions, accompanied by reduced activation of emotion arousal-related brain structures including the amygdala and the insula [11, 12]. Several meta-analyses on cognitive reappraisal in healthy and clinical populations have been published [8, 13–15]. Findings in healthy controls (HCs) indicate consistent involvement of cognitive control regions, including dorsomedial prefrontal cortex (dmPFC), dorsolateral prefrontal cortex (dlPFC), ventrolateral prefrontal cortex (vlPFC), and posterior parietal lobe during cognitive reappraisal compared to a control condition, as well as reduced activity of the left and right amygdala [13]. Kohn and colleagues [14] proposed a neural model of conscious, cognitive emotion regulation. In this model, whereas the dlPFC is related to regulation of cognitive processes such as attention, the vlPFC may not reflect the regulatory process per se, but signals salience and therefore the need to regulate. Furthermore, additional frontal areas are consistently involved in emotion regulation tasks: the anterior middle cingulate cortex (aMCC), superior temporal gyrus (STG), angular gyrus and (pre-) supplementary motor area (SMA). Furthermore, the authors proposed that the STG, angular gyrus and (pre-) SMA are involved in the execution of regulation initiated by frontal areas.

### Cognitive reappraisal in clinical populations

Zilverstand and colleagues [8] extended the literature on neural substrates underlying cognitive reappraisal to clinical populations. The authors showed that across clinical populations, individuals consistently demonstrated reduced recruitment of vlPFC and dlPFC regions, key nodes of the top-down regulatory cognitive control network in HCs. In addition, individuals with mood disorders (including MDD) showed enhanced amygdala activation during downregulation of emotion, suggesting hyperactive bottom-up responses, or reduced modulatory capacity of regulatory networks during cognitive reappraisal. In contrast, patients with anxiety disorders (including PTSD) showed reduced involvement of parietal regions, indicating impaired functionality of fronto-parietal attention networks, alongside reduced responses in the dorsal anterior cingulate cortex (dACC). Responsivity of the ACC in patients with PTSD has also been associated with symptom severity [16, 17].

In a similar line of investigation, Picó-Pérez and colleagues [15] studied the neural correlates of emotion regulation in a large sample of patients with mood or anxiety disorders and healthy individuals. The patients showed decreased activation of the prefronto-parietal network (PCC, the dmPFC, the angular gyri and the left vlPFC) in combination with increased activation in regions associated with the experience of emotions (i.e., insula, cerebellum, precentral and inferior occipital gyri) as well as in regions where activation may be the consequence of compensatory mechanisms (i.e., supramarginal gyri and superior parietal lobule). Other studies have supported these findings by demonstrating structural and functional abnormalities in the prefrontal cortex circuits related to top-down inhibitory control in MDD and PTSD [18–20].

In addition, the examination of the neural bases of volitional affect regulation in combat-related PTSD revealed that veterans with PTSD showed less recruitment of the dlPFC during cognitive regulation of affect, compared to veterans exposed to similar levels of combat stress without PTSD, suggesting altered neural activation during volitional self-regulation of negative affective states [21].

### Functional connectivity across clinical populations

Emotion dysregulation observed in MDD patients can also be considered a result of disruption of interconnected circuits. Dysfunctional connectivity of the affective salience, cognitive control, and default mode networks appears to underlie characteristic symptoms of depression, including depressed mood, anhedonia, self-rumination, and impaired concentration [22, 23]. The most frequently reported resting-state abnormalities in brain networks in patients with depression include amygdala hyperconnectivity within the salience network [24], hypoconnectivity of the frontoparietal network [25], and hyperconnectivity of the default mode network [26].

Altered emotion-related neurocircuitry has also been shown in individuals with PTSD. Sripada and colleagues [27] investigated patterns of resting-state functional connectivity (FC) of the amygdala in whole brain analyses and found that compared to HCs the veterans with PTSD showed greater positive connectivity between the amygdala and the insula, as well as between the amygdala and the hippocampus, and reduced negative connectivity between the amygdala and dorsal and rostral ACC [27]. The authors suggested that these abnormalities in emotion generation and regulation circuits may contribute to the pathophysiology of PTSD including deficits of emotion processing and emotion regulation.

PTSD and depression are highly comorbid disorders, with approximately half of patients with current PTSD also showing co-occurring depression [28]. Furthermore, both disorders are characterized by heightened levels of anxiety and depression symptoms [29]. Meta-analyses suggest shared as well as distinct neural alterations that underpin emotion regulation deficits in mood and anxiety disorders (including MDD and PTSD) [30]. However, to our knowledge, no study has directly compared these patient cohorts with respect to commonalities and differences in neural processes, including FC of emotion dysregulation in PTSD and MDD. A transdiagnostic approach targeting both PTSD and MDD should provide insight into emotion dysregulation across target groups.

The aim of the present study was to investigate the fMRI co-activation patterns during reappraisal-mediated downregulation of emotion in MDD and PTSD patients compared to healthy individuals. Moreover, the neural circuitry of emotion regulation in these clinical populations using seed-to-voxel FC was explored. We expected to replicate common alterations in frontal regulatory control regions in both patient groups as well as observe disorder-specific alterations in limbic and parietal regions and also dorsal anterior cingulum. In addition, we sought to explore associations between anxiety and depression related symptom severity across patient groups as well as associations with specific types of emotion regulation strategies (i.e., suppression and cognitive reappraisal).

## Methods

### Subjects

Thirty-five patients with MDD (age 37.3 ± 13.5) and 20 patients with PTSD (age 43.4 ± 13.0) as well as 34 healthy individuals (age 39.4 ± 12.1) without history of neurological or psychiatric disorders participated in this study. All participants were right-handed, Caucasian, had adequate knowledge of the German language, and normal or corrected to normal vision. Contraindications for MRI, pregnancy, as well as acute suicidality served as exclusion criteria for all participants. Diagnoses were based on the Diagnostic and Statistical Manual of Mental Disorders (DSM-IV-TR) criteria [31] and were confirmed by an experienced psychologist using the German version of the Structured Clinical Interview for assessment of DSM-IV-TR criteria (SCID-I) [31]. Patients were recruited through the Department of Psychiatry, Psychotherapy and Psychosomatics of the University Hospital Aachen. Additionally, patients with PTSD were recruited from a specialized outpatient clinic, the ‘Euregio Institut für Psychosomatik und Psychotraumatologie’. The extensive clinical interview was used to ensure that PTSD was the primary diagnosis. Furthermore, patients with PTSD were excluded in case of severe affective disorders based on DSM-IV criteria (major depression – moderate or severe episode; bipolar affective disorder), or substance dependence, multiple traumatic events since childhood, or acute somatic or neurologic disorders. All patients with PTSD developed symptoms after the experience of a single traumatic event. Patients with depression had stable medication for at least 1 week before participation. The data were acquired during the baseline measurements of two randomized controlled trials [5, 9]. All experiments were performed with the written informed consent of each participant and approval by the local Ethics Committee of the RWTH Aachen University Hospital, in line with the Code of Ethics of the World Medical Association (Declaration of Helsinki).

### Questionnaire acquisition and analysis

The hospital anxiety and depression scale (HADS) [32] as well as the German version of the emotion regulation questionnaire (ERQ) [32] assessed symptoms of anxiety and depression as well as emotion regulation ability. The ERQ measures respondents’ tendency to use cognitive reappraisal or expressive suppression strategies to regulate positive and negative emotions. The HADS is a self-report rating scale designed to measure anxiety and depression. Demographic and behavioral data were analyzed using a one-way ANOVA (e.g., age, ERQ, HADS) or chi-square test (e.g., gender) using STATISTICA Version 10 [33].

### Stimuli and task

Stimuli were projected onto a screen located at the back of the MRI scanner, with the stimuli viewed through an angled mirror fixed to the MRI head-coil. Stimuli were presented using the Cogent Matlab toolbox (http://www.vislab.ucl.ac.uk/cogent.php). Participants were presented 19 pictures from the International Affective Picture System (IAPS) with negative valence [34]. IAPS pictures were selected based on valence and arousal ratings resulting in a set of pictures with mean markedly negative valence (2.6 ± .33, range 2.0–3.5) and positive arousal (6.1 ± .40, 5.1–6.8). The pictures during viewing and regulation condition were balanced with respect to valence and arousal. Different randomizations were used to ensure that occurrence of each picture was similar for view and regulate.

All participants received comprehensive instructions on cognitive reappraisal strategies alongside a supervised training to apply the learned strategies to the re-evaluation of negative visual scenes prior to entering the scanner. In the scanner, each participant completed one emotion regulation run with 9 reappraise-view cycles (~ 7 min). In the ‘view’ condition, subjects were instructed to maintain the negative emotion elicited by the image. In the ‘reappraise’ condition, subjects were instructed to reappraise the content of the negative emotion by changing one’s interpretation of the negative stimulus such as imagining that (1) the situation is not as bad as it looks or (2) will get better in the future or by (3) imagining that the situation is not real or (4) taking the perspective of a professional.

**Fig. 1:**
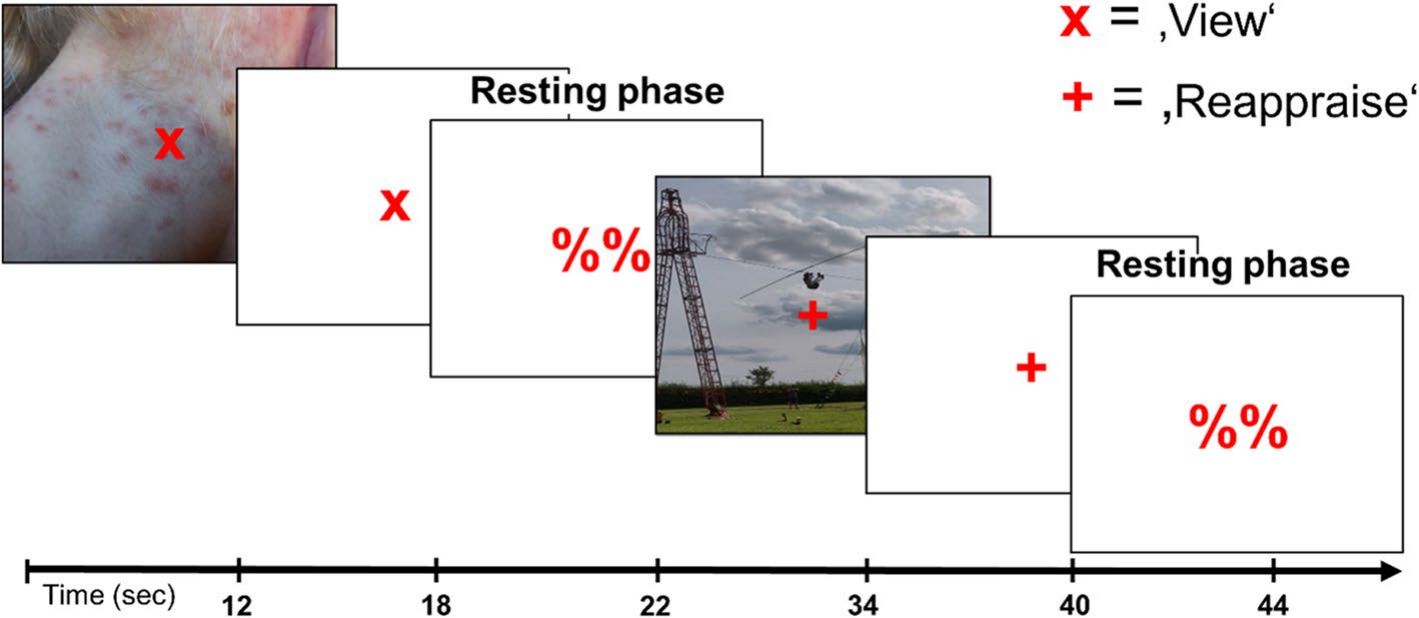
Experimental design. Participants completed 9 reappraise-view cycles with intermittent resting phases. The task was to either maintain the negative emotion elicited by a picture (‘view’ condition) or to change negative emotions of pictures by using cognitive reappraisal strategies such as imagining that (1) the situation is not as bad as it looks or (2) will get better in the future or by (3) imagining that the situation is not real or (4) taking the perspective of a professional

Preceding and following the view-reappraise cycles, participants rated their current valence and arousal level on a scale from 1 to 7 (see Fig. 1 for experimental design).

### fMRI data acquisition

The MRI scanning was performed using a 3.0 Tesla Siemens MAGNETOM Prisma scanner (Siemens Medical Systems, Erlangen, Germany) equipped with a 20-channel head coil. T2*-weighted images were obtained using echo-planar imaging with the following parameters: repetition time (TR) = 2000 ms, echo time (TE) = 36 ms, flip angle = 77 and matrix size 64 × 64. Images were acquired with 26 transverse slices in ascending interleaved order (voxel size 3 × 3 × 4 mm^3^; gap = 0.5 mm; field of view = 192 × 192 mm^2^). The first five volumes were discarded to account for T1-saturation effects. All participants were instructed to minimize movement inside the scanner and to keep eyes open and focus on the fixation cross.

### fMRI data quality assurance and preprocessing

All structural and functional data sets were examined within 48 h following recording to ensure high data quality and to enable repetition of corrupted measurements. A standard quality assurance pipeline developed and used by the Psychiatric Imaging Network Germany (PING; ping.rwth-aachen.de) was implemented. Quality parameters of the Computational Anatomy Toolbox (CAT) [35] were used for structural data. Furthermore, the Automated Quality Assurance toolbox (AQuA) [36] assured high quality of functional data. All fMRI data used in the current study had percent signal change values below 5%. Average PSC values indicate adequate data quality and did not differ between groups (HC: 2.48 ± 0.81; MDD: 2.41 ± 0.63; PTSD: 2.44 ± 0.77; *F*(2,86) = .09, *p* = .9). Preprocessing of imaging data and statistical analysis were carried out using SPM12 software (Wellcome Trust Center for Neuroimaging, London, UK) implemented in Matlab R2017b. The functional scans were first submitted to artifact correction using the ArtRepair toolbox (http://cibsr.stanford.edu/tools/ArtRepair/ArtRepair.htm), thus repairing motion/signal intensity outliers and other artifacts (including interpolation using nearest neighbors for bad scans). Slice-timing correction, head motion correction (including extraction of motion parameters), and unwarping procedures were also applied. Anatomical and functional data coregistration and spatial normalization into MNI template space were done to enable group analyses. A 128 s high-pass filter removed low-frequency drifts. Spatial smoothing was performed using an 8 mm full-width at half maximum Gaussian kernel. Head motion parameters and their temporal derivatives were included in the linear model to reduce motion artifacts. Movement parameters did not exceed 4 mm in any run.

### General linear model for the cognitive reappraisal task

For exploratory whole-brain analysis, the task-related BOLD signal changes at the subject level were estimated using a whole-brain first-level analysis based on General Linear Models (GLM) in SPM12. The first-level contrasts used for group analysis were: [reappraise > view] to detect brain networks with increased activation during cognitive reappraisal compared to baseline (view), and [reappraise < view] to identify reduced activation patterns during cognitive reappraisal compared to baseline (view). Subsequently, the contrast images of each subject were entered into a second-level model. We performed a random-effect group analysis on the contrast images using one-sample *t*-tests (*t*-tests vs 0) separately for HCs, MDD and PTSD to identify networks involved in emotion regulation for each group. T-maps for contrasts of interest [reappraise > view] represented the activation of regions involved in the cognitive control of emotion, and the opposite contrast [reappraise < view] represented regions with lower activation for regulation compared to view during this task. Second-level results were evaluated with *p*_FWE_ < .05 at voxel level for the individual groups and at *p* < .001 uncorrected voxel level and with *p* < .05 family-wise error (FWE) correction for the group difference (HC > Patients). Clusters were labelled using the automated anatomical labeling atlas 3 (AAL3) [37]. To examine whether group differences in activation were associated with differences in symptom severity of depression and anxiety (HADS) as well as differences in emotion regulation (ERQ), Pearson correlations were computed between the observed cluster in the right lateral PFC and questionnaire data. Correlation analyses were corrected for multiple comparisons (4 tests: α = 0.05/4 = 0.0125).

### ROI analysis

We focused our analysis on a priori regions previously implicated in the cognitive reappraisal literature [14, 15]. The MarsBaR toolbox (version 0.44) [38] was used to create ROI spheres of 10 mm radius centered on the peak coordinates provided in previous meta-analyses [14, 15]. The selected ROIs were: 1) the posterior cingulate cortex (PCC), extending into the precuneus (− 4,-38,2); 2) the bilateral dorsomedial prefrontal cortex (dmPFC) [left_dmPFC: (− 6,32,48) and right_dmPFC (16,20,50)]; 3) the bilateral angular gyrus (AG) [left_AG: (− 42,-72,34) and right_AG (60,-54,40)]; 4) the left ventrolateral prefrontal cortex (vlPFC) [left_vlPFC; − 54,36,-2)]; 5) bilateral inferior frontal gyrus (IFG) [left_IFG: (− 42,22,-6) and right_IFG (50,30,-8)]; 6) left middle temporal cortex (MTC; 38, 22, 44); 7) bilateral precentral gyrus (PG) [left_PG: (− 44,10,46) and right_PG (42–16 34)] and 8) (pre-) supplementary motor area (SMA) (− 2,14,58). Finally, the mean ROI beta values were extracted from the contrast maps of all subjects using MarsBaR. Beta values were compared between HCs, MDD and PTSD patients using a one-way ANOVA (*p* < .05) followed by post-hoc Tukey’s Honest Significant Difference (HSD) to correct for multiple comparisons. Post-hoc tests were done in STATISTICA Version 10 [33].

### Connectivity analyses

To investigate FC group differences, we conducted a task based as well as voxel-to-voxel connectivity analysis using the FC toolbox CONN (www.nitrc.org/projects/conn, RRID:SCR_009550). The data were preprocessed using the default preprocessing pipeline for volume-based analysis which includes realignment and unwarping of functional data, slice- timing correction, outlier identification, direct segmentation, and normalization as well as functional smoothing with a Gaussian kernel of 8 mm full width half maximum (FWHM).

#### Seed-to-voxel connectivity analysis

To investigate task-based seed-to-voxel connectivity, a generalized psychophysiological interaction (gPPI; McLaren and colleagues [39]) was computed. The gPPI is the more recent version of the PPI that can accommodate more than two task conditions. Furthermore, by modeling the entire experimental space it may have greater sensitivity and specificity than standard PPI. Signal variance that correlated with the seed region during the regulation compared to view condition (‘reappraise – view’) was investigated. The right inferior frontal gyrus (pars triangularis and opercularis) was chosen as a seed region. Second-level results were evaluated at *p* < .005 uncorrected voxel level and with *p* < .05 FWE-correction at the cluster level.

#### Voxel-to-voxel connectivity

Within network changes during cognitive reappraisal (contrast between reappraise vs. view) as well as group differences of voxel-to-voxel connectivity were investigated. Voxel-to-voxel connectivity is a measure of network centrality at each voxel and characterizes the strength of the connectivity pattern between each voxel and the rest of the brain (root mean square of the correlation coefficient values) [40]. Second-level results were evaluated at *p* < .005 uncorrected voxel level and with *p* < .05 FWE-correction at the cluster level.

## Results

### Demographics and self-report data

**Fig. 2:**
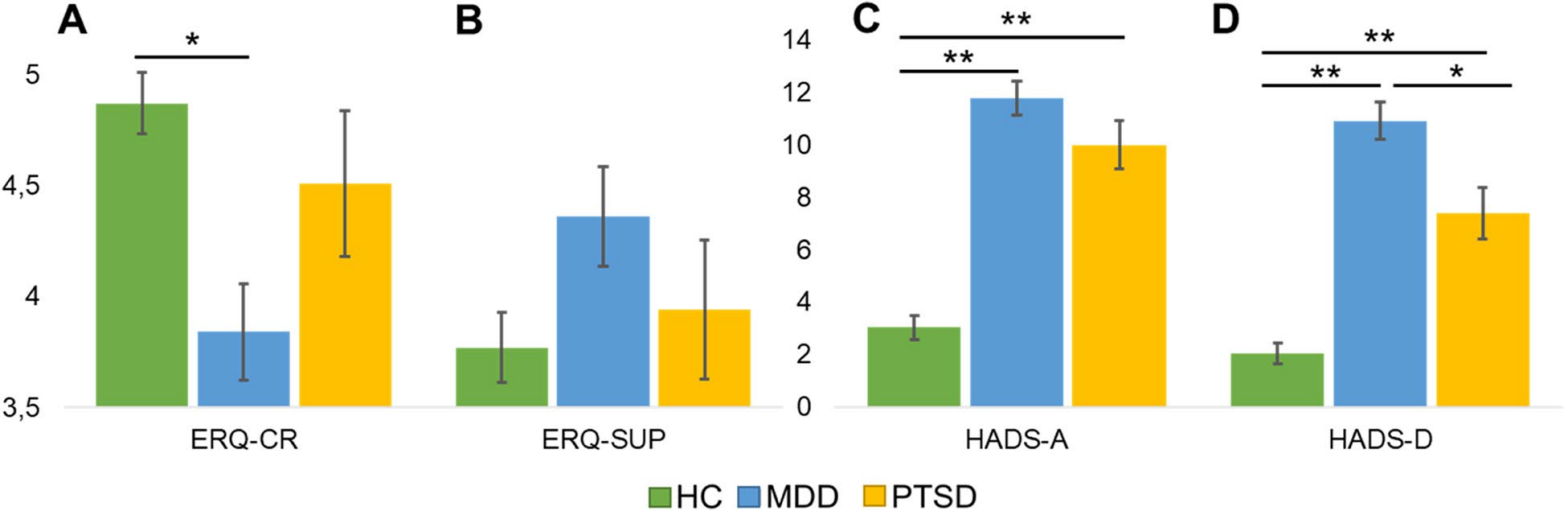
Mean rating scores of clinical scales in healthy individuals, patients with depression and PTSD. **A** Patients with depression used significantly less cognitive reappraisal than healthy individuals. **B** The three groups showed a similar tendency to use suppression strategies. C MDD and PTSD patients showed higher levels of anxiety compared to healthy controls. **D** MDD patients had the highest scores of depressive symptoms. Error bars denote standard errors of the mean. Significance: * = *p* < .01; ** = *p* < .001. Abbreviations: ERQ: Emotion regulation questionnaire (CR: cognitive reappraisal, SUP: suppression); HADS: Hospital anxiety and depression scale (A: anxiety, D: depression); HC: Healthy controls; MDD: Major depressive disorder; PTSD: Post-traumatic stress disorder

Analyses of demographic data indicated no differences in age between the three groups (*F*(2, 86) = 1.408, *p* = .25). Furthermore, the three groups did not differ with regard to gender (*Χ* ^2^(2, *N* = 89) = .0173, *p* = .99). One-way ANOVAs investigated the main effect of group on emotion regulation (ERQ) and symptom severity of depression and anxiety (HADS) (see Table 1 and Fig. 2). There was a significant main effect of group for cognitive reappraisal (ERQ-CR; *F*(2, 80) = 6.6, *p* = .002). Post-hoc comparisons with the Tukey HSD test revealed that patients with depression reported less use of cognitive reappraisal than the control group (*p* = .002). The test revealed no group differences in the following comparisons: i) Controls vs PTSD (*p* = .15) and ii) MDD vs PTSD (*p* = .58). A separate ANOVA revealed no main effect of group for suppression strategies (ERQ-SUP; *F*(2, 86) = 2.079, *p* = .13).

**Table 1:** Demographic and clinical characteristics

	**Controls (** ***n*** **= 34)**		**MDD (** ***n*** **= 35)**		**PTSD (** ***n*** **= 20)**				
	*n*	%	*n*	%	*n*	%	*X* ^2^		*p*
Gender (male)	17	50	18	51.4	10	50	.017		.99
	*M*	*SD*	*M*	*SD*	*M*	*SD*	*F*	*df*	*p*
Age	39.38	12.1	37.34	13.5	43.40	13.0	1.41	2, 86	.25
ERQ_CR	4.87	0.81	3.84	1.31	4.51	1.47	6.6	2, 80	.002
ERQ_SUP	3.77	0.93	4.36	1.34	3.94	1.4	2.08	2, 80	.13
HADS_A	3.03	2.59	11.76	3.86	10	4.07	56.2	2, 83	.001
HADS_D	2.03	2.34	10.91	4.25	7.4	4.42	49.02	2, 84	.001

*ERQ* Emotion regulation questionnaire (subscales: *CR* Cognitive reappraisal, *SUP* Expressive suppression), *HADS* Hospital anxiety and depression scale (subscales: *A* Anxiety, *D* Depression), *MDD* Major depressive disorder, *PTSD* Post-traumatic stress disorder

Furthermore, there were main effects of group for both subscales of the HADS (HADS-Anxiety (*F*(2,83) = 56.2, *p* < .001); HADS-Depression (*F*(2,84) = 49.06, *p <* .001)). Healthy individuals had lower scores on the anxiety subscale than patients with depression (*p* < .001) and PTSD (*p* < .001). Both clinical groups showed similar levels of anxiety (*p* = .18). Healthy controls also obtained lower scores on the depression subscale than MDD (*p* < .001) and PTSD (*p* < .001) patients. However, patients with depression showed higher levels of depressive symptoms than patients with PTSD (*p* < .01).

### Monitoring task success

**Fig. 3:**
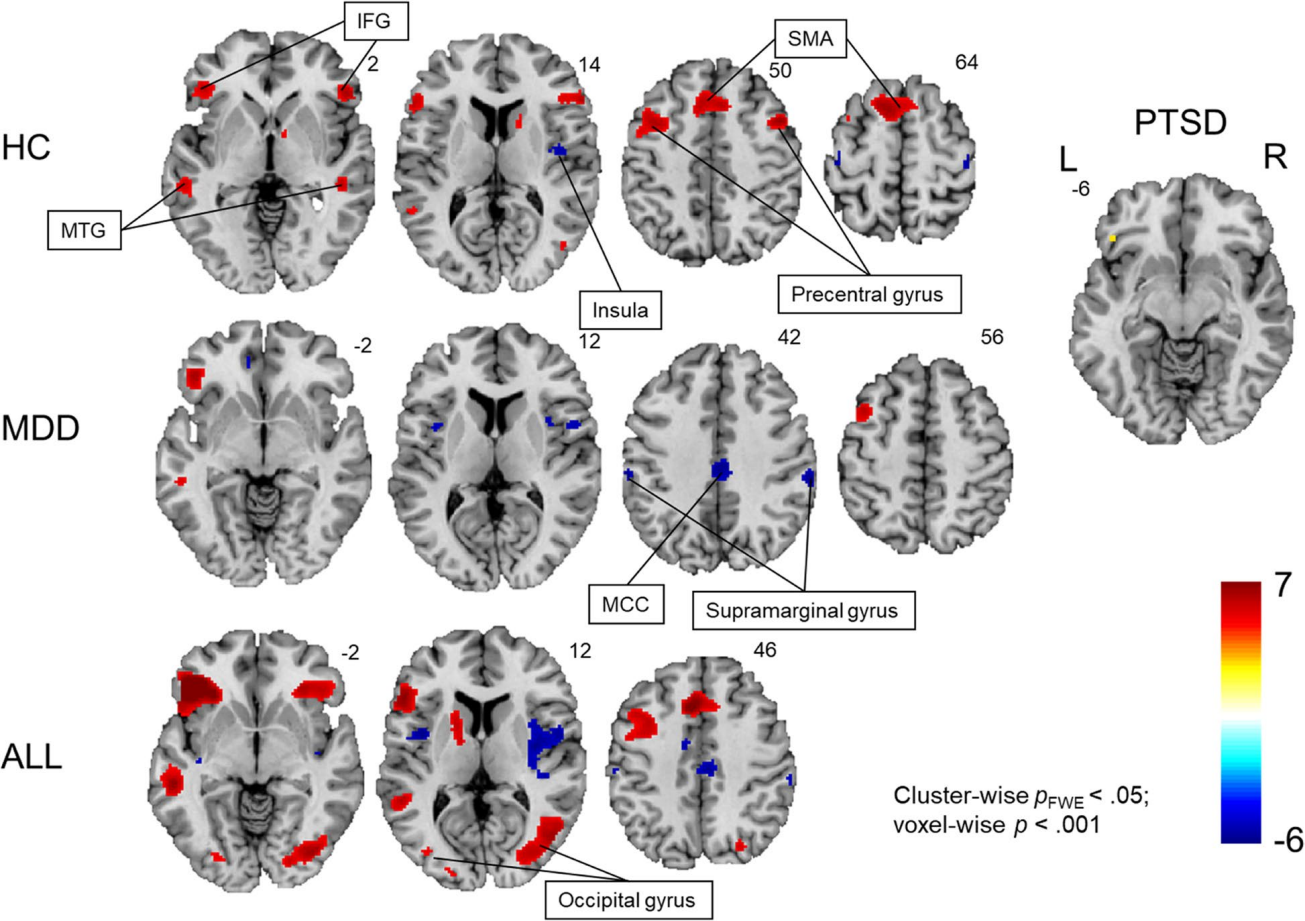
Whole-brain functional brain maps depicting increased activation (reappraise > view) and decreased activation (view > reappraise) during cognitive reappraisal for healthy controls (HCs), patients with major depressive disorder (MDD) and patients with post-traumatic stress disorder (PTSD). Activation in the left IFG was found in all groups while activation during cognitive reappraisal was most extensive in HCs

To ensure participants’ adherence to the task we used different measures. All participants received sufficient training on cognitive reappraisal to make sure they understood the task. Furthermore, following the scanning sessions, participants were asked to their applied cognitive reappraisal strategies on a subsample of pictures. This way we could conclude that all participants used strategies related to cognitive reappraisal. However, as behavioral, and neural effects may dissociate [41], we also focused on neural outcomes. Our whole brain functional maps (Fig. 3) as well as the first-level brain maps showed that participants recruited brain areas (e.g., lateral PFC) consistently related emotion regulation and cognitive reappraisal, respectively [14].

### Whole-brain fMRI analysis

To examine group effects of reappraising compared to viewing negative stimuli, two contrasts were examined with one sample *t*-tests for each individual group on the second level. First, the contrast ‘reappraise > view’ represented the activation of regions involved in the cognitive control of emotion in the group of HCs. Second, the opposite contrast ‘reappraise < view’ represented brain regions that were less active during reappraisal compared to ‘view’ (see Fig. 3 and Additional file 1: Appendix 1 for overview of results). These analyses revealed increased activation during cognitive reappraisal for HCs in the bilateral IFG (pars triangularis), bilateral middle frontal gyrus, left precentral gyrus, bilateral SMA, bilateral temporal gyrus, right caudate, and right thalamus. Lower activations during cognitive reappraisal were found in the right insula. The same analysis in patients with MDD revealed only activation in the left IFG (pars triangularis), left middle frontal gyrus, right cerebellum, left medial segment of the superior frontal gyrus and left insula during cognitive reappraisal. However, patients with MDD showed significantly lower activation for reappraisal compared to ‘view’ in the right middle cingulate gyrus, right rolandic operculum, bilateral insula, left Heschl’s gyrus and right supramarginal gyrus (see Fig. 3). In the PTSD group cognitive reappraisal was related to a significant cluster in the left IFG (pars orbitalis and triangularis).

**Fig. 4:**
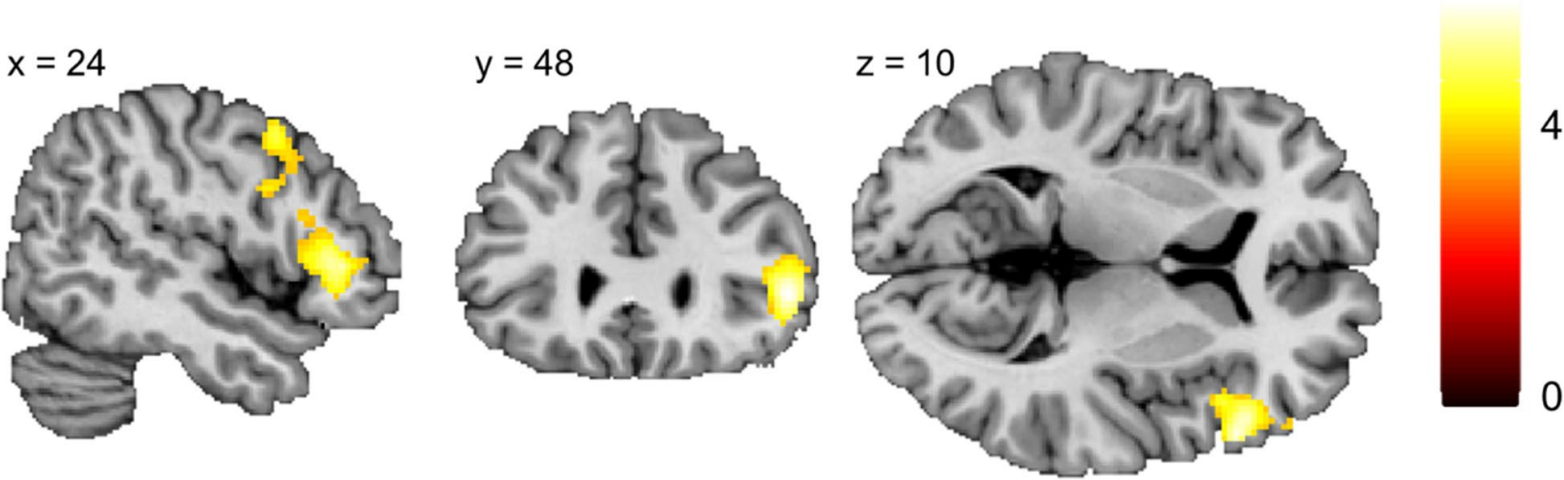
Higher brain activation in the right triangular and opercular parts of the IFG in healthy individuals compared to patients (MDD and PTSD) for the contrast reappraise > view (*T*_Peak_ = 4.96, extend threshold = 783 voxels). Results are corrected at *p* < .001 (unc.) with cluster-correction at *p*_FWE_ < .05

Furthermore, the same model was used to evaluate group differences between HCs and patients with MDD and PTSD. HCs had greater activation compared to patients (MDD + PTSD) in a cluster covering (according to AAL3) the right triangular (46.4%) and opercular (23.5%) parts of the IFG as well as the right precentral gyrus (17.4%) [(56 20 8), *T*_peak_ = 4.96] (see Fig. 4). *T*-tests between HCs and MDD revealed a similar cluster in the right precentral gyrus (30.5%), the right opercular (29.3%) and triangular (26.4%) part of the IFG [(56 20 8), *T*_peak_ = 4.7)]. At the selected threshold there were no significant differences between HCs and patients with PTSD [(52 26 4), *T*_peak_ = 4.3, *p*_FWE_ = .08] as well as between patients with MDD and PTSD. Furthermore, the three groups did not differ regarding lower activation during cognitive reappraisal compared to viewing.

To examine the relation of dysfunctional neural patterns during cognitive reappraisal and behavioral parameters, the cluster comprising the group difference (HC > Patients) was extracted and Pearson correlations between ROI mean beta values and ERQ as well as HADS results were computed. Significance was assessed based on a corrected threshold for the number of comparisons (*n* = 4) of α = .0125. Significant negative correlations of HADS anxiety and depression scales with beta values (HADS-Anxiety (*r* = −.38, *N* = 81, *p* < .001) and HADS-Depression (*r* = −.34, *N* = 81, *p* < .01)) suggested that a higher clinical severity of anxiety or depression was related to less recruitment of the right IFG during cognitive reappraisal. A similar analysis revealed no significant association between mean beta values of the right lateral PFC ROI and the self-report of cognitive reappraisal (ERQ-CR; *r* = .12, *N* = 81, *p* = .27) or suppression (ERQ-supression; *r* = −.07, *N* = 81, *p* = .52).

### ROI analysis

**Fig. 5:**
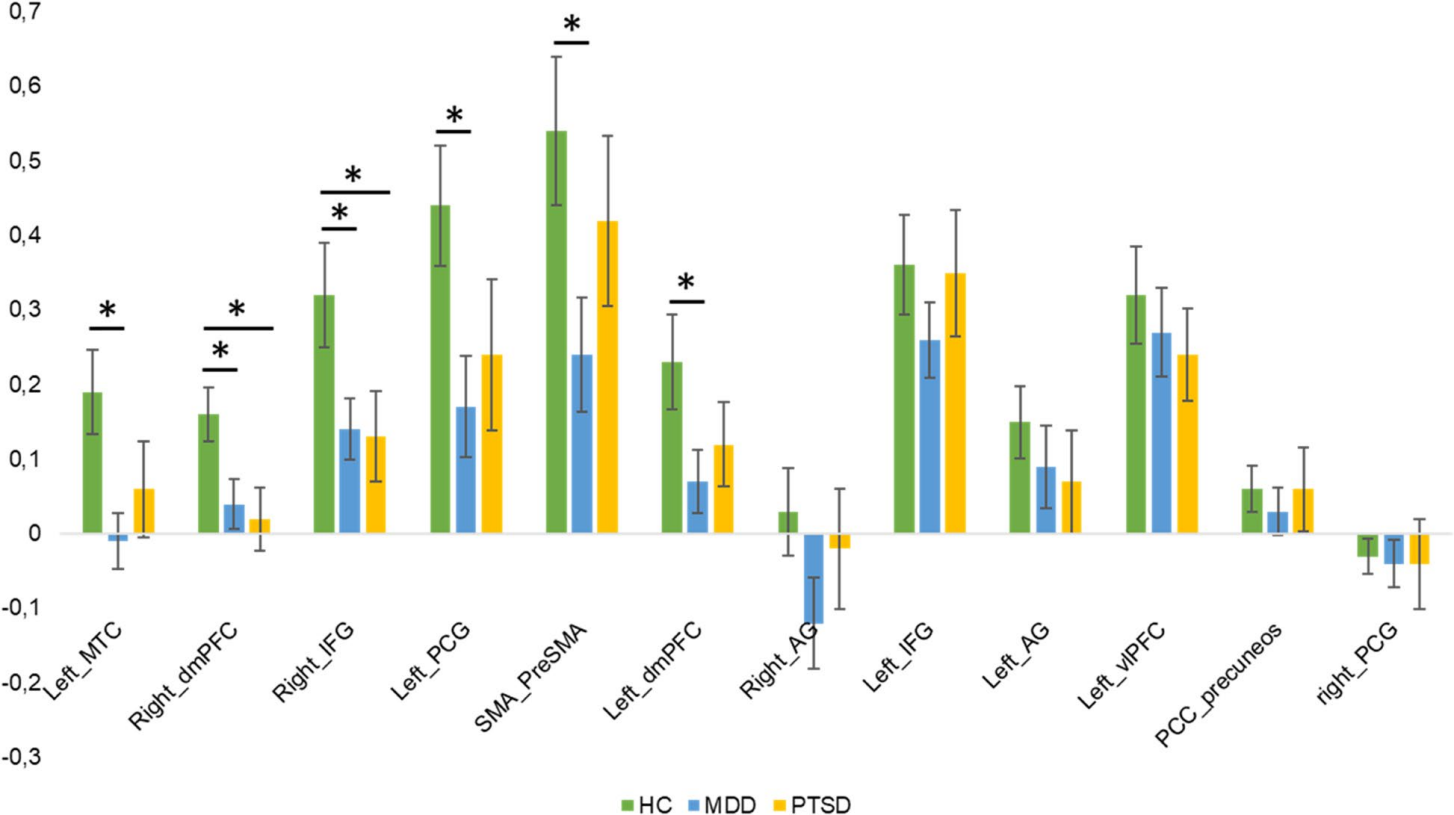
Comparison of ROI peak activity between groups (healthy controls (HCs), major depressive disorder (MDD) and post-traumatic stress disorder (PTSD)). Beta values were extracted for the contrast ‘reappraise > view’ for each region of interest. Significantly higher beta values (Tukey’s correction) for HCs compared to MDD patients were found in the left middle temporal cortex, bilateral dorsomedial PFC, right inferior frontal gyrus, and supplementary motor cortex. Furthermore, beta values were significantly higher for HCs compared to PTSD patients in the right dorsomedial PFC and right IFG. Error bars display standard errors of mean

For the ROI analysis, a priori regions previously been associated with cognitive reappraisal were selected [14, 15]: bilateral angular gyrus, bilateral IFG, bilateral dorso-medial prefrontal cortex, bilateral precentral gyrus, the left middle temporal cortex (MTC), the left ventrolateral prefrontal cortex, and the bilateral supplementary motor area/pre-supplementary motor area. A 3 (Group [MDD, PTSD, HC]) × 12 (ROI) ANOVA was computed to investigate differences in mean ROI values for the contrast ‘reappraise > view’. The ANOVA revealed a significant main effect of group for the left middle temporal cortex, right dorsomedial prefrontal cortex, right inferior frontal gyrus and left pre-central gyrus (Fig. 5).

Post-hoc tests (HSD) showed that HCs had higher activation in the left MTC, PCG as well as dmPFC and SMA compared to patients with depression and higher activation in the right dmPFC and IFG compared to both patients with depression and PTSD.

### Connectivity analysis

To evaluate task-related differences in FC between HCs and patients with MDD and PTSD, we conducted a seed-to-voxel connectivity analysis using the right IFG as a priori region of interest. To further investigate the functional organization of the brain during the cognitive reappraisal task without a priori assumptions, we conducted a voxel-to-voxel connectivity analysis.

#### Seed-to-voxel connectivity analysis

Using the right IFG (pars triangularis and opercularis) as seed, we investigated the effect of cognitive reappraisal compared to viewing of negative pictures on brain system interactions. For the between-conditions contrast (reappraise > view), separate *t*-tests were computed for HCs as well as MDD and PTSD patients. HCs exhibited increased FC between the rIFG seed and the left cerebellum, left occipital fusiform gyrus, left lingual gyrus and precuneus (cluster: − 26 -48 -26, size = 808, *p*FWE < .0001; cluster: − 22 -96 + 08, size = 334, *p*FWE = .004 and cluster: − 16 -80 + 20, size = 311, *p*FWE = .007) as well as the right pre/postcentral gyrus and the right superior parietal lobule (cluster: + 16–30 + 44, size = 309, *p*FWE = .007). In addition, increased FC was found in an anterior network cluster comprised of the right superior frontal gyrus and the middle frontal gyrus (cluster: + 24–18 + 60, size = 245, *p*FWE = .028). Patients with MDD demonstrated increased FC between the rIFG and the bilateral cerebellum as well as the left occipital fusiform gyrus (cluster: − 02 -72 -34, size = 908, *p*FWE < .0001). Reduced FC emerged between the rIFG and two clusters comprising the left middle frontal gyrus (cluster: − 32 + 12 + 42, size = 544, *p*FWE < .0001), the precuneus, left lateral occipital cortex, left middle frontal gyrus and posterior cingulate gyrus (cluster: − 32 + 12 + 42, size = 544, *p*FEW < .0001). PTSD patients showed increased FC between the rIFG seed and a antero-central network comprised of the bilateral paracingulate gyrus, the anterior cingulate gyrus, and bilateral medial and superior frontal gyrus (cluster: + 08 + 46–04, size = 268, *p*FWE = .0018; cluster: + 00 + 48 + 22, size = 238, *p*FWE = .03).

Table 2 contains between-group connectivity estimates. Compared to HCs, the PTSD group showed increased FC of the rIFG with the left frontal orbital cortex, left temporal pole, left inferior frontal gyrus (pars opercularis), left frontal operculum, and left insular cortex but less coupling between rIFG and the right lateral occipital cortex. PTSD patients showed greater functional coupling of the rIFG and the precuneus, the anterior and posterior cingulate gyrus, the bilateral paracingulate gyrus, the bilateral superior frontal gyrus, the left frontal orbital cortex, the left frontal gyrus, the left temporal pole, the left superior frontal gyrus and left insula compared to MDD patients. The HC > MDD comparison showed no significant differences in connectivity during cognitive reappraisal.

**Table 2:** Seed-to-voxel connectivity for right IFG (pars triangularis + opercularis) seed during cognitive reappraisal (reappraise > view). Threshold was *p* < .005 voxel-level and FWE-cluster correction *p* < .05

	Peak MNI coordinates [mm]			Extent [voxel]	Peak *t*-values
	x	y	z		
** Brain regions**					
**PTSD > HC **					
Frontal orbital cortex (l)	−42	24	−12	529	−5.49
Temporal pole (l)					
Inferior frontal gyrus (l)					
Frontal operculum (l)					
Insular cortex (l)					
** PTSD > MDD**					
Precuneus	−06	−38	42	1338	6.53
Posterior cingulate gyrus					
Paracingulate gyrus (b)	00	48	18	634	4.23
Anterior cingulate gyrus					
Superior frontal gyrus (b)					
Frontal orbital cortex (l)	−46	24	−16	414	5.32
Temporal pole (l)					
** HC > MDD**					
none of the voxels					
survived correction					

#### Voxel-to-voxel connectivity

**Fig. 6:**
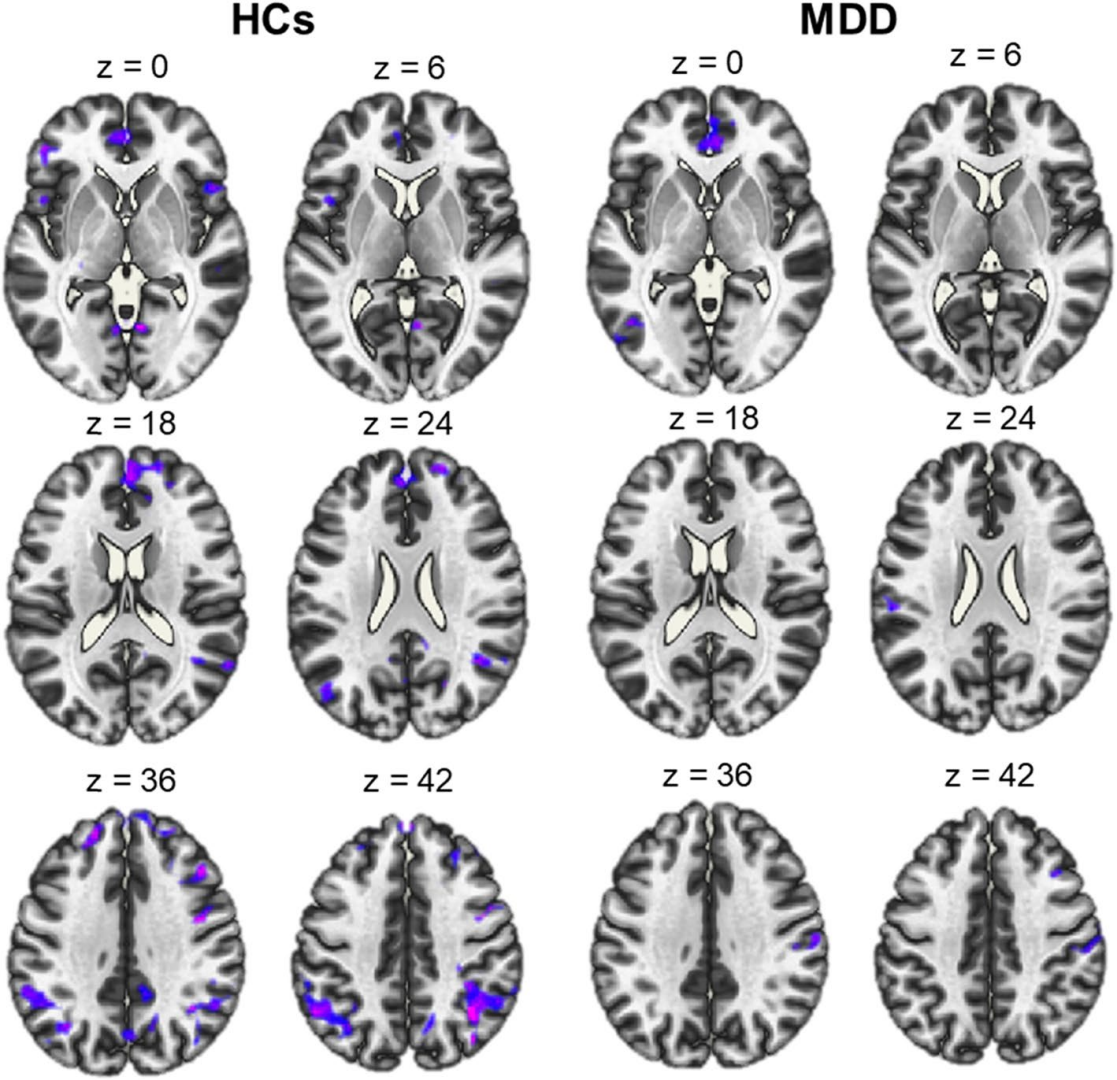
Voxel-to-voxel connectivity in healthy controls (HCs) and patients with major depressive disorder (MDD). The whole-brain voxel-based connectivity revealed reduced connectivity of neural networks during the cognitive reappraisal task (contrast between ‘reappraise vs. view’). The significant threshold was *p* < .005 uncorrected voxel level and with *p* < .05 FWE-correction at the cluster level. HCs showed a wide-spread neural network distribution of voxel-to-voxel connectivity during the task. In contrast, patients with MDD showed less pattern of activation during the task. No clusters in the post-traumatic stress disorder group survived correction

As a data driven measure of functional network organization during the emotion regulation task, we investigated voxel-to-voxel connectivity. HCs showed a global reduction of voxel-to-voxel connectivity in a widespread network during cognitive reappraisal. The largest effect of voxel-to-voxel connectivity was present in a right-lateralized anterior network, which included the middle frontal gyrus, the paracingulate gyrus, the superior frontal gyrus, and the anterior cingulate gyrus. Patients with MDD showed reduced voxel-to-voxel connectivity in an anterior network including the supplementary motor cortex, the left paracingulate gyrus and bilateral superior frontal gyrus. Finally, the analysis showed no significant voxel-to-voxel connectivity patterns in PTSD patients during cognitive reappraisal (see Fig. 6).

Differences between groups were identified using paired *t*-tests (*p* < .005 unc. at voxel level and *p* < .05 FWE-corrected at cluster level; 124-voxel cluster threshold; Additional file 1: Appendix 2). Compared to HCs, PTSD patients exhibited significantly higher voxel-to-voxel connectivity values in a posterior network including the precuneus, posterior cingulate gyrus, bilateral lingual gyrus, bilateral intracalcarine cortex, bilateral cuneal cortex, right supracalcarine cortex and the bilateral occipital cortex. The MDD group also showed a significantly higher voxel-to-voxel connectivity compared to HCs in two clusters in the precentral gyrus. The comparison between clinical groups showed that PTSD patients exhibited increased voxel-to-voxel connectivity relative to patients with MDD in the right lateral occipital cortex, right angular gyrus, right middle temporal gyrus, right supramarginal gyrus, and right superior temporal gyrus.

## Discussion

The identification of pathophysiological mechanisms common to psychiatric disorders as well as mechanisms unique to specific psychiatric disorders are critical for elucidating the biological mechanisms underlying psychiatric symptoms and developing targeted treatment protocols [42, 43]. The purpose of this study was to identify differences in brain activation patterns between patients with psychiatric disorders and HCs during cognitive regulation of negative emotions. More specifically, we focused on the comparison of patients with MDD and PTSD. In this study, we employed a classical emotion regulation paradigm to identify emotion regulation networks common and specific to HCs as well as patients with MDD and PTSD by contrasting reappraisal of negative pictures with natural viewing of negative pictures. Both clinical groups showed higher scores of anxiety and depression compared to HCs, and with a higher self-reported severity of depression in MDD compared to PTSD patients. On the neurobiological level, exploratory analyses showed that MDD and PTSD patients had less right-lateralized activation of the inferior, middle and superior frontal gyrus during cognitive reappraisal (reappraise vs view) compared to HCs. Importantly, this cluster in the right IFG was negatively correlated with the scores within the hospital anxiety and depression scale (HADS), suggesting that less recruitment of right IFG may be associated with greater severity of anxiety as well as depressive symptomatology. Furthermore, our seed-to-voxel and voxel-to-voxel analyses confirmed group differences in connectivity during cognitive reappraisal. To our knowledge this is the first study directly comparing focal neural activation and FC patterns in HCs, MDD and PTSD patients.

### Comparison with the existent literature on neural correlates of cognitive reappraisal in clinical populations

Our whole brain fMRI analysis revealed significant activation of the bilateral inferior frontal gyrus (*pars triangularis*), bilateral temporal, middle frontal, and superior frontal gyrus during cognitive reappraisal. Previous studies consistently report that cognitive reappraisal engages a prefronto-parietal network which exerts top-down control on limbic areas [14, 15, 44]. Accordingly, our results are largely consistent with the literature.

In terms of seed-to-voxel fMRI connectivity, HCs exhibited increased FC between the rIFG seed and clusters distributed along posterior and frontal regions. Consistent with previous studies, our findings suggested the existence of several large-scale networks that are co-activated during cognitive reappraisal. Morawetz and colleagues [45] identified four clusters of co-activation patterns during emotion-generative and emotion-regulatory processes to brain-networks underlying prominent psychological functions and evaluated their specificity in relation to emotion regulation. Increased connectivity between rIFG and superior and middle frontal gyri as well as with the precuneus and the left lingual gyrus overlaps with a network which plays an intermediary role in reappraisal by integrating information from prefrontal and subcortical areas to generate and regulate emotional responses.

Patients with depression and PTSD displayed reduced activation of the vIPFC (a crucial node of the emotion regulatory network) during downregulation of negative emotion [8]. Wang and colleagues [7] examined the neural mechanisms of self-related reappraisal in Chinese MDD outpatients and found that depressed individuals exhibited diminished activation in left IFG when detachment strategies were adopted (subjects should view the situation as fake or unreal and detach themselves from the situation). Contrary to some previous reports, we found that patients showed reduced activation, especially in the right-lateralized IFG, but not in the left-homologous region. However, this functional hemispheric asymmetry has also been found by other investigators reporting a greater role for the right hemisphere in the processing of negative affect in HCs [46, 47] and impairments in clinical conditions. Furthermore, Wager and colleagues [48] showed that right vlPFC activity predicted drops in self-reported negative emotion, and that this relationship was independently mediated by separate pathways through the amygdala and the ventral striatum, thus stressing the importance of the right vlPFC for emotion regulation. Interestingly, neuroimaging studies report that activation in the vlPFC is strongly lateralized based on the type of stimuli processed [49]. Studies addressing the differences between upregulation and downregulation of negative emotions report that upregulation engaged primarily left-lateralized prefrontal regions, whereas downregulation engaged bilateral prefrontal regions [47].

In our study, we used strategies aimed at downregulating negative stimuli. Contrasting reappraisal versus view showed bilateral vlPFC activation in the control group. This is consistent with the pattern of right-lateralized activation for downregulation of negative emotions described by Ochsner and colleagues [50]. The altered right vlPFC activation in both patient groups implies an impaired cognitive control capacity of bottom-up systems, such as the amygdala that appraise the affective properties of stimuli. In general, the vlPFC - especially right vlPFC - is thought to play an important role in response inhibition as a particular form of executive control [51]. Studies have highlighted the importance of the right hemisphere during cognitive reappraisal, engaging greater activation when the stimulus is more difficult and more cognitively demanding to reappraise [52]. The correlation of right vlPFC activation with depressive and anxious symptomatology further supported the findings of the vlPFC as a key region involved in cognitive reappraisal across mood and anxiety disorders.

In addition to impairments across psychopathologies, Zilverstand and colleagues [8] reported deficits specific for mood and anxiety disorders. For patients with MDD, they found hyperactivity of the amygdala during downregulation of negative emotions, a finding in line with enhanced bottom-up responses and reduced top-down modulatory ability. Our ROI analysis indicated disorder-specific deficits for patients with MDD during cognitive reappraisal in the dmPFC, SMA, left middle temporal cortex and left precentral gyrus. These areas have been related to the execution of reappraisal, suggesting that a deficit in signaling the need for regulation in the vlPFC leads to less effective reappraisal [14], a pattern specific to patients with MDD in our sample.

Regarding the neural correlates of emotion regulation deficits in PTSD, in addition to the reduced activation in the right IFG, the group of PTSD patients showed hypoactivation in the right dmPFC. Previous studies have focused on the structure, neurochemistry, and function of the amygdala, medial prefrontal cortex, and hippocampus in PTSD. Contrary to the hyperresponsiveness of the amygdala during emotion regulation, the medial prefrontal cortex appears to be volumetrically smaller and is hyporesponsive during symptomatic states and the performance of emotional cognitive tasks in PTSD [53]. This finding is consistent with our results.

### Seed-to-voxel and voxel-to-voxel functional connectivity

FC analyses further investigated the importance of the group difference in the right IFG for cognitive reappraisal. In a seed-to-voxel fMRI connectivity analysis using the right IFG as seed region, PTSD patients exhibited altered FC compared to HCs and MDD patients during reappraisal. Compared to HCs, PTSD patients showed increased FC of the rIFG with the left frontal orbital cortex, the left temporal pole, the left inferior frontal gyrus (pars opercularis), and the left frontal operculum. Interestingly, our results indicated altered connectivity predominantly in the PTSD group, suggesting higher FC in a fronto-temporo-parietal network. Higher connectivity in this network may result from a compensatory mechanism, taking into account that regions are important for emotion regulation and more specifically cognitive control mechanisms [54]. Although it may be speculative, this fronto-temporo-parietal hyperconnectivity may also reflect the use of suppression strategies that rely on engagement of this network. Furthermore, abnormalities on fronto-limbic connectivity have been the most discriminant feature for classification of PTSD based on the resting-state connectivity between the prefrontal and limbic regions [55].

In addition, PTSD patients showed greater FC than MDD patients in regions including the precuneus, the anterior and posterior cingulate gyrus and the bilateral paracingulate gyrus. This finding is consistent with reports of PTSD patients showing increased FC in the anterior cingulate gyrus as a core component of the salience network [56]. This may be related to the role played by the ACC in monitoring and appraisal of the external environment, reciprocally connecting brain regions to regulate stressor-related autonomic nervous system activity [57]. The PCC is also implicated in stress neural circuit [58], and researchers have found anatomical and functional change of this region in patients with PTSD [59, 60].

To investigate more general connectivity group differences, we employed a voxel-to-voxel connectivity analysis as a whole-brain voxel-based connectivity measure that represents how well connected any given voxel is to the rest of the gray matter voxels in the brain. Compared to the conventional seed-based connectivity analyses, voxel-to-voxel connectivity does not require a priori knowledge for the selection of the regions of interest [40]. In the present work, the comparison between HCs vs PTSD and PTSD vs MDD during cognitive reappraisal indicated voxel-to-voxel connectivity abnormalities in PTSD patients. Previous studies have demonstrated impaired within- and between networks FC in PTSD. For example, Akiki and colleagues [61] report hypoactivity of the default mode network (DMN) and central executive network (CEN) that are putatively destabilized by an overactive and hyperconnected salience network (SN), which appears to be associated with an inefficient DMN-CEN modulation [61]. For the DMN network, we found that the group of PTSD patients differed from HCs in the activation of core regions such as the posterior cingulate cortex and precuneus. It has been suggested that DMN hypoactivity is associated with a deficit in the processing of autobiographical memory and self-references which may be a core underlying mechanism regarding PTSD symptomatology [62].

### Limitations

There are some limitations of the current study. First, the smaller sample size of PTSD compared to HCs and patients with MDD may have impeded the detection of significant differences between these groups. In addition, PTSD patients presented with relatively mild symptoms and whereas most individuals in the MDD group were treated on the ward, a high percentage of PTSD patients was treated in an outpatient clinic. In addition, different subtypes of emotion regulation in PTSD may complicate interpretation of the data. A previous review described a model that includes these two types of emotion dysregulation in PTSD [63]. In this model, reexperiencing/hyperarousal reactivity is viewed as a form of emotion dysregulation that involves abnormally low activation in medial anterior brain regions, mediated by failure of prefrontal inhibition of limbic regions. In contrast, the dissociative subtype of PTSD is described as a form of emotion dysregulation that involves emotional overmodulation mediated by midline prefrontal inhibition of the same limbic regions. Both types of modulation are involved in a dynamic interplay and lead to alternating symptom profiles in PTSD [63]. Furthermore, we selected a homogenous set of IAPS pictures based on valence and arousal ratings, however, this was not entirely possible. Hence, neural responses to specific images may also be a function of differences in arousal measures. Lastly, psychopharmacological treatment regimens differed between groups. Accordingly, we cannot exclude the possibility that the observed differences in neural activation patterns between groups may be attributable to drug effects.

## Conclusions

Presented results underscored the importance of the lateral PFC for cognitive reappraisal across mood and anxiety disorders. Patients with MDD and PTSD showed reduced activation within the right IFG. Supporting a key role of this region in psychopathology, reduced right IFG activity predicted a greater severity of anxiety or depression in both patients with MDD and PTSD. However, seed-based FC of the right IFG and voxel-to-voxel connectivity patterns during cognitive reappraisal in PTSD patients were significantly different from those of HCs and MDD patients. In addition to hypoactivation of right IFG found in MDD and PTSD patients, these FC patterns suggested the underlying emotional processing of stimuli may show specific patterns for mood and anxiety disorders. Our findings underscore the importance of the lateral PFC for cognitive reappraisal and further suggest that the right IFG may be a suitable target for fMRI-based neurofeedback or other neuromodulatory interventions that may contribute to an improvement of symptoms.

## Supplementary Information

**Additional file 1: Appendix 1.** Regions demonstrating significant activations (reappraise > view) or deactivations (view > reappraise) in healthy controls, patients with MDD and patients with PTSD. **Appendix 2.** Between group differences in intrinsic connectivity as a measure of a voxel-analysis during cognitive reappraisal (reappraise > view).

## Data Availability

The data that support the findings of this study are available from RWTH Aachen University Hospital, but restrictions apply to the availability of these data, which were used under license for the current study, and so are not publicly available. Data are however available from the authors upon reasonable request and with permission of RWTH Aachen University Hospital.
