# Supplementary material for: Transdiagnostic alterations in neural emotion regulation circuits – neural substrates of cognitive reappraisal in patients with depression and post-traumatic stress disorder
Source: BMC Psychiatry. 2022 Mar 8;22:173. doi: 10.1186/s12888-022-03780-y (PMC8905757; doi:10.1186/s12888-022-03780-y)
Supplement: Supplementary file 1 — Additional file 1: Appendix 1. Regions demonstrating significant activations (reappraise > view) or deactivations (view > reappraise) in healthy controls, patients with MDD and patients with PTSD. Appendix 2. Between group differences in intrinsic connectivity as a measure of a voxel-analysis during cognitive reappraisal (reappraise > view). [file 12888_2022_3780_MOESM1_ESM.docx]

**Supplementary material**

Appendix 1

Regions demonstrating significant activations (reappraise > view) or deactivations (view > reappraise) in healthy controls, patients with MDD and patients with PTSD.

| **HC (activation)** | MNI coordinates [mm] | | |  |  |  |  |
| --- | --- | --- | --- | --- | --- | --- | --- |
| Brain region | x | y | z | Extent [voxel] | peak *t*-value |  |  |
| Left MFG | -36 | 4 | 58 | 418 | 7.08 |  |  |
| Right MFG | 46 | 6 | 52 | 207 | 6.97 |  |  |
| Left IFG_tri | -44 | 28 | -2 | 490 | 6.63 |  |  |
| Left SFG | -14 | 10 | 66 | 1283 | 6.57 |  |  |
| Right IFG_tri | 48 | 26 | -2 | 502 | 6.54 |  |  |
| Right MTG | 48 | -36 | 2 | 70 | 5.90 |  |  |
| Left MTG | -58 | -36 | 2 | 139 | 5.79 |  |  |
| Left Thal_VL | -14 | -8 | 6 | 41 | 5.54 |  |  |
| Right TMP | 48 | 18 | -20 | 15 | 5.53 |  |  |
| Right Thal_VA | 8 | -4 | 2 | 7 | 5.35 |  |  |
| Right IFG_tri | -50 | 28 | 28 | 9 | 5.33 |  |  |
| Left TMP | -44 | 20 | -26 | 3 | 5.32 |  |  |
| Right MOG | 44 | -74 | -26 | 19 | 5.18 |  |  |
| Right CAU | 16 | 10 | 14 | 14 | 5.17 |  |  |
| Left MFG | -30 | 50 | 22 | 11 | 5.14 |  |  |
| Right IFG_oper | 44 | 10 | 36 | 41 | 5.13 |  |  |
| Right Thal_VL | 16 | -6 | 8 | 4 | 5.00 |  |  |
| Right MFG | 32 | 28 | 50 | 2 | 4.98 |  |  |
| Left IOF | -30 | -84 | -8 | 3 | 4.92 |  |  |
|  |  |  |  |  |  |  |  |
| **HC (deactivation)** | MNI coordinates [mm] | | |  |  |  |  |
| Brain region | x | y | z | extent [voxel] | peak *t*-value |  |  |
| Right POG | 46 | -22 | 62 | 24 | 5.89 |  |  |
| Left POG | -44 | -22 | 62 | 24 | 5.89 |  |  |
| Right Pins | 34 | -14 | 18 | 24 | 5.89 |  |  |
| Right POG | 46 | -22 | 62 | 24 | 5.89 |  |  |
|  |  |  |  |  |  |  |  |
| **MDD (activation)** | MNI coordinates [mm] | | |  |  |  |  |
| Brain region | x | y | z | extent [voxel] | peak *t*-value |  |  |
| Left IFG_tri | -46 | 30 | -4 | 24 | 5.89 |  |  |
| Left MFG | -40 | 8 | 58 | 24 | 5.89 |  |  |
| Right cerebelum | 36 | -58 | -28 | 24 | 5.89 |  |  |
| Left MTG | -56 | -40 | -2 | 24 | 5.89 |  |  |
| Right cerebelum | 10 | -80 | -18 | 13 | 5.06 |  |  |
|  |  |  |  |  |  |  |  |
| **MDD (deactivation)** | MNI coordinates [mm] | | |  |  |  |  |
| Brain region | x | y | z | extent [voxel] | peak *t*-value |  |  |
| Right SMG | 62 | -34 | 44 | 87 | 6.291 |  |  |
| Left SMG | -62 | -32 | 42 | 23 | 6.085 |  |  |
| Right MCgG | 2 | -26 | 44 | 215 | 5.852 |  |  |
| Right CO | 52 | 2 | 10 | 55 | 5.632 |  |  |
| Right Ins | 36 | 4 | 12 | 18 | 5.299 |  |  |
| Left CO | -40 | 0 | 12 | 15 | 5.211 |  |  |
| Left HeschlG | -38 | -22 | 2 | 5 | 5.164 |  |  |
| Right Pcu | 10 | -70 | 42 | 4 | 5.012 |  |  |
| Right SLP | 46 | -44 | 58 | 2 | 4.994 |  |  |
| Left AG | -10 | 40 | -2 | 7 | 4.987 |  |  |
|  |  |  |  |  |  |  |  |
| **PTSD (activation)** | MNI coordinates [mm] | | |  |  |  |  |
| Brain region | x | y | z | extent [voxel] | peak *t*-value |  |  |
| Left IFG_tri | -42 | 28 | -0 | 26 | 5.11 |  |  |
|  |  |  |  |  |  |  |  |
| **PTSD (deactivation)** | MNI coordinates [mm] | | |  |  |  |  |
| Brain region | x | y | z | extent [voxel] | peak *t*-value |  |  |
| none of the voxels survived correction | | | | | |  |  |
| Clusters reported at *p* < .05 FWE-corrected. | | | | | |  |  |

IFG_tri: inferior frontal gyrus (triangular part); IFG_orb: IFG pars orbitalis; MFG: middle frontal gyrus; MTG: middle temporal gyrus; SFG: superior frontal gyrus; STG: superior temporal gyrus; SMC: supplementary motor cortex; CO: Central operculum; MCG: middle cingulate gyrus; AI anterior insula; PO parietal operculum; Thal_VL (VA): Thalamus_ventral lateral (ventral anterior); MCC: Middle cingulate & paracingulate gyri; ROL: Rolandic operculum; HES: Heschl’s gyrus; SMG: SupraMarginal gyrus; CAU: Caudate nucleus; PCUN 'Precuneus

Appendix 2

Between group differences in intrinsic connectivity as a measure of a voxel-analysis during cognitive reappraisal (reappraise > view).

| **HC > PTSD** | Peak MNI coordinates [mm] | | | Extent [voxel] | peak *t*-value |
| --- | --- | --- | --- | --- | --- |
| Brain region | x | y | z |  |  |
| r Lateral Occipital Cortex, r Superior Parietal Lobule, r Angular Gyrus, r Supramarginal Gyrus, r Postcentral Gyrus, precuneus cortex | 36 | -58 | 42 | 1026 | -7.22 |
| precuneus cortex, posterior Cingulate Gyrus, r cuneal cortex r lateral occipital cortex | 12 | -66 | 40 | 1024 | -6.35 |
| b Paracingulate Gyrus, b Superior Frontal Gyrus, anterior Cingulate Gyrus | 2 | 52 | 18 | 330 | -4.27 |
| r Frontal Orbital Cortex, r Insular Cortex, r Temporal Pole, r Inferior Frontal Gyrus pars triangularis, r Frontal Operculum Cortex, r Inferior Frontal Gyrus pars opercularis | 42 | 18 | -14 | 278 | -5.41 |
| r Middle Frontal Gyrus, r Precentral Gyrus, r Superior Frontal Gyrus | 32 | 20 | 38 | 270 | -5.76 |
| b Lingual Gyrus, r Intracalcarine Cortex, precuneus cortex, Vermis, r Supracalcarine cortex, Vermis | 8 | -62 | 6 | 237 | -5.01 |
| l Lateral Occipital Cortex, l Superior Parietal Lobule | -30 | -54 | 40 | 142 | -4.51 |
| r Middle Frontal Gyrus | 32 | 42 | 40 | 128 | -4.44 |
|  |  |  |  |  |  |
| **HC > MDD** |  |  |  |  |  |
| b Precentral Gyrus | -14 | -30 | 60 | 203 | -4.23 |
| Cerebellum | 34 | -68 | -40 | 163 | -4.89 |
|  |  |  |  |  |  |
| **PTSD > MDD** |  |  |  |  |  |
| r Lateral Occipital Cortex, r Angular Gyrus | 38 | -66 | 46 | 383 | 4.88 |
| r Middle Temporal Gyrus temporooccipital, r Middle Temporal Gyrus posterior division, r Supramarginal Gyrus posterior division, r Superior Temporal Gyrus posterior division | 56 | -38 | 04 | 188 | 4.77 |
| Cerebellum | -12 | 70 | -50 | 165 | 6.45 |
| precuneus cortex, r Lateral occipital cortex superior division | 10 | -76 | 46 | 141 | 3.79 |
| Voxel threshold *p* < .005. | | | | | |

*Abbreviations*: sLOC: lateral occipital cortex; FP: Frontal Pole; SPL: Superior Parietal Lobule; AG: Angular Gyrus; PC: posterior cingulate; AC: Cingulate Gyrus; MidFG: Middle Frontal Gyrus; pSMG: posterior supramarginal Gyrus; LG: Lingual Gyrus; PaCiG: Paracingulate Gyrus; FOrb: Frontal Orbital Cortex; ICC: Intracalcarine Cortex; r = right; l = left; b = bilateral.
